# CUP-1 Is a Novel Protein Involved in Dietary Cholesterol Uptake in *Caenorhabditis elegans*

**DOI:** 10.1371/journal.pone.0033962

**Published:** 2012-03-27

**Authors:** Victor J. Valdes, Alejandro Athie, Laura S. Salinas, Rosa E. Navarro, Luis Vaca

**Affiliations:** Departamento de Biología Celular y del Desarrollo, Instituto de Fisiología Celular, Universidad Nacional Autónoma de México, Ciudad Universitaria, México DF, México; University of South Florida College of Medicine, United States of America

## Abstract

Sterols transport and distribution are essential processes in all multicellular organisms. Survival of the nematode *Caenorhabditis elegans* depends on dietary absorption of sterols present in the environment. However the general mechanisms associated to sterol uptake in nematodes are poorly understood. In the present work we provide evidence showing that a previously uncharacterized transmembrane protein, designated Cholesterol Uptake Protein-1 (CUP-1), is involved in dietary cholesterol uptake in *C. elegans*. Animals lacking CUP-1 showed hypersensitivity to cholesterol limitation and were unable to uptake cholesterol. A CUP-1-GFP fusion protein colocalized with cholesterol-rich vesicles, endosomes and lysosomes as well as the plasma membrane. Additionally, by FRET imaging, a direct interaction was found between the cholesterol analog DHE and the transmembrane “cholesterol recognition/interaction amino acid consensus” (CRAC) motif present in *C. elegans* CUP-1. *In-silico* analysis identified two mammalian homologues of CUP-1. Most interestingly, CRAC motifs are conserved in mammalian CUP-1 homologous. Our results suggest a role of CUP-1 in cholesterol uptake in *C. elegans* and open up the possibility for the existence of a new class of proteins involved in sterol absorption in mammals.

## Introduction

Sterols are essential for eukaryotic cells and many cellular processes depend directly or indirectly on them. In the free-living nematode *Caenorhabditis elegans*, cholesterol is involved in important biological functions, including growth control and reproduction. Its supply is essential for the production of sterol-derived hormones (e.g. dafachronic acids/gamravali), which regulate development and mating behavior [1]–[4]. Cholesterol has been also proposed as a protein modifier of important signaling molecules, modulating its activity and transport [5], [6].

Nematodes are auxotrophic for sterols; they cannot synthesize lanosterol and squalene, key metabolites in sterol biosynthesis [1]. Consequently, absorption of dietary sterols is absolutely essential for the survival of *C. elegans*. [2], [7]–[9]. In their natural environment, worms obtain sterols from exogenous sources such as bacterial membranes and animal and plant debris [1], [10]. In laboratory conditions, cholesterol is added to the media to support normal growth and reproduction. Nematodes can survive with trace amounts of cholesterol, although effects like reduced offspring and stress resistance as well as growth defects are observed for several generations [11]–[14]. In complete cholesterol deprivation, more severe phenotypes are observed, including aberrant somatic gonad development, alterations in germ-cell proliferation as well as locomotion and molting defects [3], [15], [16] If cholesterol deprivation is maintained, a general developmental arrest is observed at early larval stages with subsequent death [17].

Even though no specific tissues for fat storage have been identified in *C. elegans*, cholesterol staining with filipin or growing animals in the presence of fluorescent cholesterol analogs has revealed that some cells and structures are enriched in cholesterol, i.e. the pharynx, the excretory gland cell, the nerve ring, the intestine and phasmid socket cells in the tail. Oocytes are also rich in sterols, where cholesterol is essential to support embryonic development. [12], [14], [18]

Despite the vital importance of sterols in *C. elegans*, the precise molecular mechanisms responsible for cholesterol uptake and distribution are poorly understood. One protein proposed to be involved in cholesterol uptake in *C. elegans* is the LDL receptor-like protein-1 (LRP-1) [16]. Mutations in the *lrp-1* gene lead to phenotypes that share some aspects of cholesterol deprivation such as shedding inability and growth arrest, but have no effect on body size and fertility. Furthermore, *lrp-1* expression is restricted to the apical surface of the epithelium involved in body cuticle formation [19]. Due to its collagen-rich structure, it is unlikely that sterols passively cross the body cuticle to enter the worm. It seems that LRP-1 is involved in molting by promoting cuticle degradation through secretion or activation of proteases and collagenases, rather than sterol uptake [16], [20].

In mammals, cholesterol uptake from the bloodstream is accomplished by the receptor-mediated endocytosis of low-density lipoprotein (LDL)-cholesterol particles. In *C. elegans*, a similar system is responsible for cholesterol uptake in oocytes, where RME-2, a member of the LDL receptor superfamily, mediates the endocytosis of vitellogenins (cholesterol-rich proteins) from the body cavity [18], [21]. However, it is clear that the *C. elegans* vitellogenin/RME-2 cholesterol-uptake system cannot be responsible for all cholesterol absorption as cholesterol uptake in hermaphrodite larvae starts earlier than vitellogenin expression and males -that do not express vitellogenins at all- are able to uptake cholesterol. Furthermore, neuronal cells -that do not accumulate vitellogenins- are rich in cholesterol [13].

In humans, a crucial step in cholesterol metabolism is its exit from late endosomes. Mutations in NPC1 (Niemann-Pick type C protein) result in accumulation of cholesterol in lysosomes that ultimately leads to neurodegeneration [22]. This supports a model where NPC1 acts as a molecular pump involved in the intracellular transport of cholesterol from endosome to ER [23], [24]. In *C. elegans*, two NPC1 related genes have been identified: *ncr-1* and *ncr-2* [25]. *Ncr-1* is expressed in tissues with high levels of cholesterol (e.g. intestine), while *ncr-2* expression is restricted to a particular set of neurons [26]. Lack of NCR-1 results in growth delay under cholesterol deprivation, probably as a result of alterations in steroid hormone processing [25]–[27]. Absence of NCR-1 and NCR-2 results in the constitutive establishment of dauers; a larval stage that provides resistance and it is only observed under hostile conditions [25], [26].

Although, there is evidence of the intracellular mechanism of cholesterol transport in *C. elegans*, very little is known about the molecular entities and the mechanisms responsible for dietary cholesterol uptake.

Numerous proteins that interact with cholesterol contain a “cholesterol recognition/interaction amino acid consensus” (CRAC) motif in their sequences. This CRAC motif is characterized by the pattern L/V-X_(1–5)_-Y-X_(1–5)_-R/K [28]. Structural evidence shows that CRAC is a cholesterol binding site where Tyr directly interacts with the polar 3′OH-group of cholesterol, Leu/Val interact with the hydrophobic side chain, and Arg/Lys form the cholesterol-binding pocket [29]. CRAC motifs are present in many proteins involved in transport, metabolism and regulation of cholesterol, including the peripheral-type benzodiazepine receptor, Apo A1, G-protein coupled receptors, caveolin-1 and hedgehog among many others [30].

**Figure 1:**
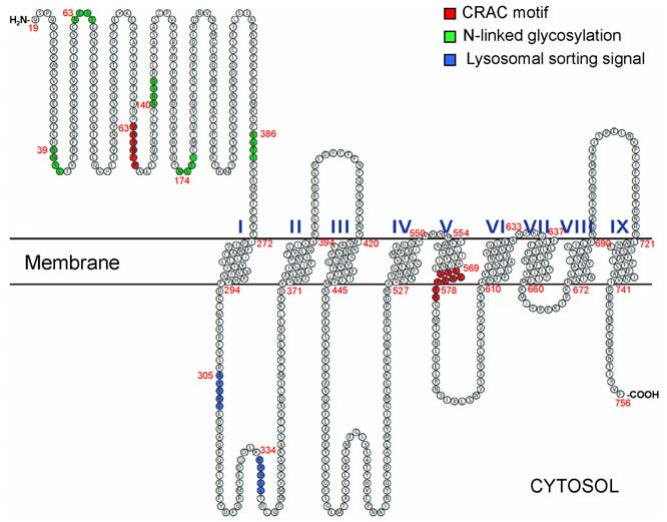
Predicted topological structure of *C. elegans* Cholesterol Uptake Protein-1 (CUP-1). A topology model built from CUP-1 hydrophobicity profile is shown predicting nine transmembrane domains with an extracellular N-terminus and an intracellular C-terminus. The conserved CRAC motifs (L/V-X_(1–5)_-Y-X_(1–5)_-R/K) in the extracellular portion and in the fifth transmembrane domain are highlighted in red. Potential YXXØ/DXXLL endocytic motifs are shown in blue. N-linked glycosylation sites in the extracellular N-terminus are shown in green. The first 17 amino acids at the N-terminus are predicted to be a signal peptide and were excluded from this figure. Red numbers indicate amino acid residue. Roman numbers indicate transmembrane domains.

A careful sequence analysis of a previously uncharacterized *C. elegans* gene, ZK721.1/*tag-130* (temporarily assigned gene-130) [31], showed the presence of two conserved CRAC motifs in its sequence: one in the extracellular N-terminus and another in the fifth-transmembrane domain (Figure 1). In the present work, we have identified cholesterol interaction between the transmembrane CRAC motif and cholesterol analogs. We also showed that animals lacking this protein are hypersensitive to cholesterol availability in the media and their capacity to uptake and distribute cholesterol is diminished. Due to these findings we named this protein Cholesterol Uptake Protein-1 (CUP-1) and we suggest it plays an important role in dietary cholesterol uptake in *C. elegans*.

## Results

### Lack of *cup-1* results in fertility problems when grown in low-cholesterol conditions

**Figure 2:**
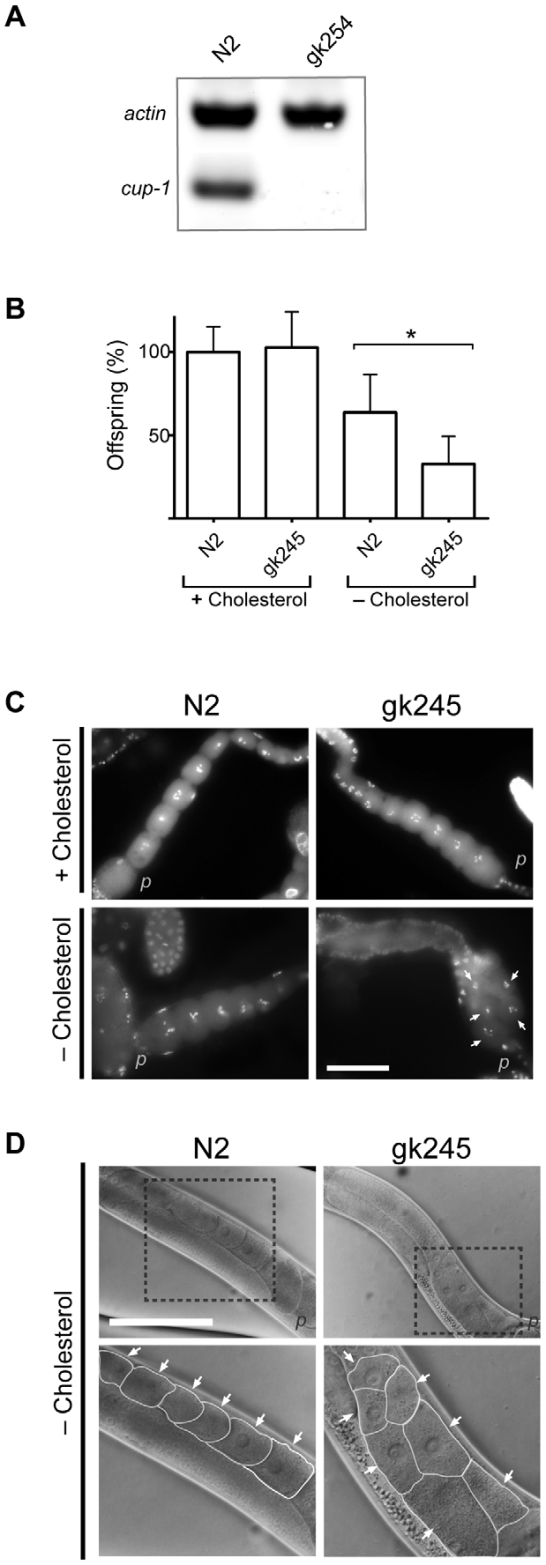
Oogenesis defects in *cup-1* mutant animals grown in low-cholesterol conditions. (A) RT-PCR of the *cup-1(gk245)* animals shows absence of *cup-1* mRNA. N2 is the wild-type strain and the *actin* gene was used as positive control. (B) Total offspring percentage of wild-type and *cup-1(gk245)* animals grown with cholesterol supplementation or in low-cholesterol conditions. Data were normalized to the average offspring of wild-type with cholesterol as 100%. Error bar: sem; * p<0.001. (C) DAPI staining of gonads of wild-type and *cup-1(gk245)* animals grown with cholesterol supplementation (upper panel) or in low-cholesterol conditions (lower panel). Arrows indicate nuclei where alterations in distribution of oocytes inside the gonad were observed. Scale bar 50 µm. (D) Nomarski imaging of the mid section of wild-type and *cup-1(gk245)* animals grown in low-cholesterol conditions. Rectangles show zoom areas to the gonads (lower panels). Arrows point oocytes where the double-line array of oocytes is observed in the mutant animals. Membranes are highlighted in white for clarity. *p*: proximal. Scale bar 100 µm.

Since CRAC motifs have been identified as cholesterol-binding sites, we first investigated the role of CUP-1 in *C. elegans* cholesterol metabolism. To do this, we used *cup-1(gk245)* animals, a strain carrying a 771 bp deletion comprising the *cup-1* promoter as well as the first exon. Absence of *cup-1* mRNA was assessed by RT-PCR (Figure 2A). Since complete cholesterol deprivation results in early death in *C. elegans* [14], nematodes were grown with trace amounts of cholesterol (mainly from agar) to identify whether increased sensitivity to cholesterol limitation was present in the *cup-1* mutant animals.

The first parameter evaluated was offspring number: as expected, wild-type animals grown in low-cholesterol media showed a 25% decrease in offspring number but *cup-1(gk245)* animals showed a 72% reduction (Figure 2B, Table 1) compared to animals grown in cholesterol-supplemented media. Additionally, closer examination of the gonads from *cup-1(gk245)* animals by Nomarski and fluorescence microscopy showed that 47% of the subjects presented a double-line array of oocytes (Figure 2C and 2B) albeit, 24% of the subjects showed no alterations in oocytes morphology. Moreover, we observed a 60% reduction in oocyte number in 1 day-old cup-*1(gk245)* adult hermaphrodites grown in low-cholesterol conditions and a five-fold increase in embryonic lethality in comparison to wild-type animals (Table 1). Interestingly, sperm cell number revealed no alterations either with or without cholesterol supplementation (Table 1, Figure S1 A). Importantly, no alterations were seen in wild-type animals grown in low-cholesterol conditions or in *cup-1(gk245)* grown with normal amounts of cholesterol in any of the parameters evaluated (Figure 2B, Table 1).

**Table 1:** Hypersensitiveness of *cup-1(gk245)* animals to cholesterol availability.

	Cholesterol	Total Offspring(mean ± SD)	Sperm Number (mean ± SD)	Oocyte Number (mean ± SD)	Embryo Lethality (% ± SD)	Larval Development (% ± SD)	Dauer(% ± SD)
N2	+	250±13	110.2±11.5	5.8±0.73	0.28±0.27	100±0.0	2.8±0.3
	−	189±38	117.8±10.8	5.5±0.78	0.32±0.45	98.6±1.8	8.0±0.5
gk245	+	272±35	111.0±11.2	5.2±0.85	0.57±0.46	98.9±1.3	0.5±0.2*
	−	70±21*	110.3±10.2	3.3±1.1*	3±1.0*	78.2±16.4*	2.2±0.6*

Wild-type (N2) and *cup-1* mutant animals (*gk245*) were grown with cholesterol supplementation or in low-cholesterol conditions and phenotypes were evaluated in F2 progeny. For total offspring, embryos and larvae were quantified from individual nematodes for six consecutive days. Sperm and oocyte numbers were obtained from DAPI stained gonads. Embryo lethality is defined as the percentage of non-hatched embryos after 24 h. Larval development is defined as the number of adults divided by the total number of L1 larvae. Dauer percentage was evaluated by larvae survival to SDS treatment after 15 days of growth at 25°C. For Offspring, Embryo Lethality and Larval Development, n>70 from three independent determinations. For Sperm and Oocyte Number, n>40. Dauer, n>1500.

* p<0.01.

To corroborate that the phenotype was specific to the lack of CUP-*1*, we performed RNAi against *cup-1* in wild-type animals (Figure S1 B). Similar alterations in oocyte distribution to the ones observed in the *cup-1* mutant were observed in 42% of RNAi subjects grown in low-cholesterol conditions. Additionally, a reduction in oocyte number was also observed in RNAi animals (Figure S1 C). In summary, *cup-1* defective animals, recapitulate hallmark phenotypes associated with hypersensitivity to cholesterol limitation affecting oogenesis, such as reduced fertility, alterations in oocyte number and distribution, as well as increased embryo lethality when grown in low-cholesterol conditions.

### Lack of *cup-1* affects animal growth, mobility and stress resistance

**Figure 3:**
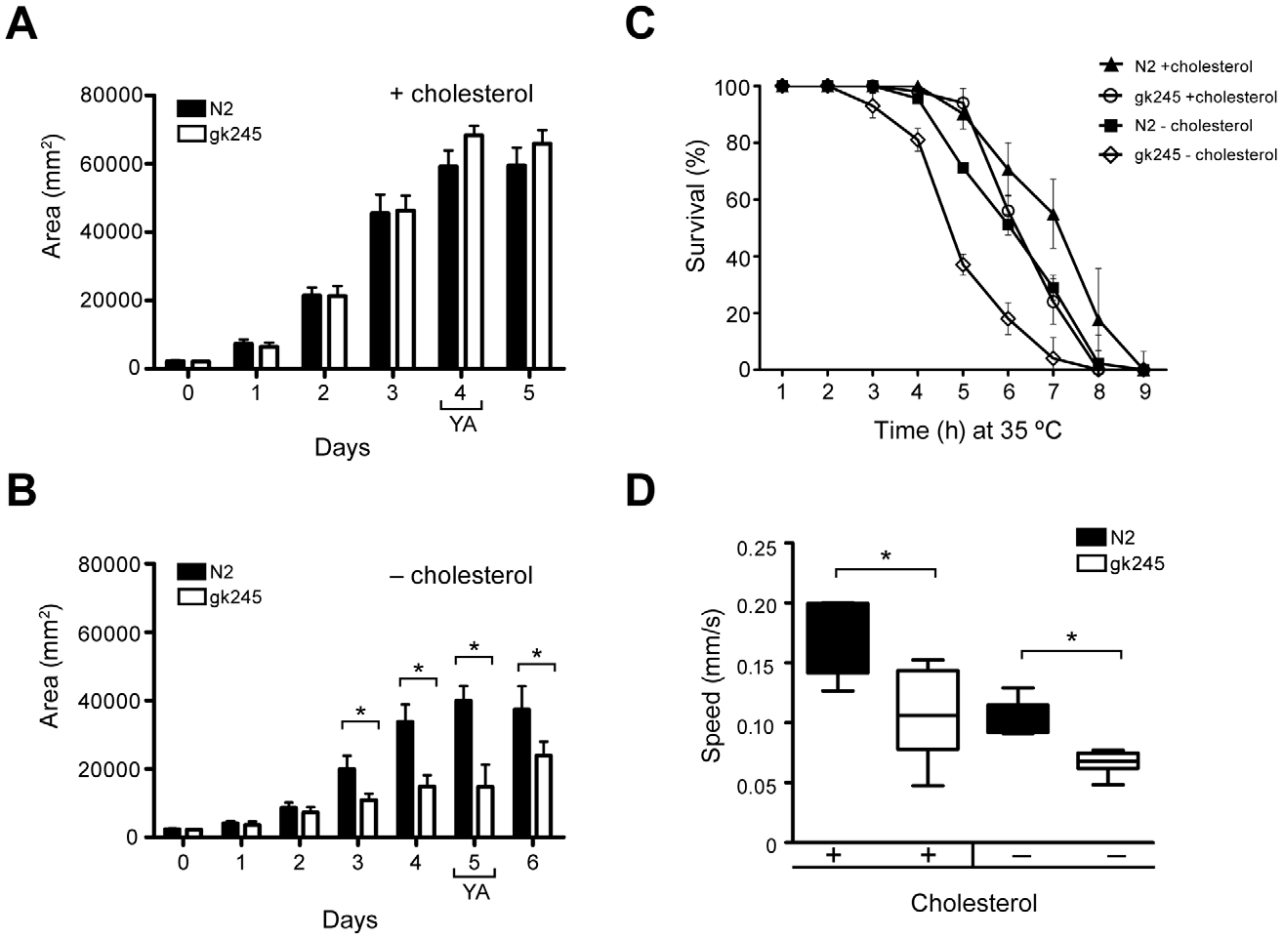
The *cup-1* mutant animals were hypersensitive to cholesterol limitation. (A and B) *cup-1(gk245)* animals were smaller in low-cholesterol conditions. Animal size was estimated from worm area of L1 larvae (day 0) to wild-type adults (N2) and *cup-1(gk245)* animals grown with cholesterol supplementation (A) or in low-cholesterol conditions (B). YA: young adults; error bar: sem; n>100; * p<0.001. (C) *cup-1* mutant animals were more sensitive to heat stress. Survival of worms at 35°C was evaluated in wild-type and *cup-1(gk245)* animals growing with or without cholesterol supplementation in the media. Error bar: sem. n>40. (D) Speed was severely affected in *cup-1(gk245)* animals. Worm speed was estimated from 10 min video recordings of over 20 animals in plates without food and grown with cholesterol supplementation or in low-cholesterol conditions. Boxes represent first to third quartiles around the median. Bars: min. and max. values. n>300 tracks; * p<0.001.

Cholesterol limitation has also been associated with a decrease in *C. elegans* size [12], [15]. Thus, we evaluated this parameter measuring nematode area during development. In normal cholesterol supplementation, there were no alterations in size between *cup-1(gk245)* and wild-type animals (Figure 3A). In low-cholesterol conditions, the daily increase in animal size was not significantly affected for the first 2 days after hatching; however, growth of the *cup-1(gk245)* animals essentially ceased by day 3, resulting in a 63% reduction in adult size at day 5 compared to wild-type (14,800 µm^2^ *vs* 40,000 µm^2^) (Figure 3B). A 22% reduction in the percentage of L1 larvae that reached adulthood was also observed in *cup-1(gk245)* animals grown in low-cholesterol conditions (Table 1). Nevertheless, no alterations in morphology were detected during embryo or larval development in the *cup-1(gk245)* animals that reached adulthood (Figure S2 A and B), except for a delay of 4–5 h in the L4-to-adult transition.

It has been reported that cholesterol metabolism is important for dauer larvae formation [2], [3], [13], [25], [26]. Therefore we decided to evaluate the role of CUP-1 in this larval stage. We observed a 78% reduction of dauers in the *cup-1(gk245)* mutant animals (Table 1). However, this dauer formation deficiency was independent of cholesterol availability. This may be attributed to low cholesterol levels that could have a negative impact in cuticle formation and/or stress resistance rather than a direct relationship with dauer induction.

Cholesterol metabolism is also associated with stress resistance in nematodes since a decrease in lipid storages has a negative impact on stress resistance [32], [33]. Thus, to evaluate stress responsiveness of *cup-1(gk245)* animals, one-day-old adult hermaphrodites were subjected to heat stress for several hours, and median lethal time (LT_50_) was estimated. A higher sensitivity to heat exposure was observed in *cup-1(gk245)* animals grown in low-cholesterol conditions with a 23.8% thermoresistance reduction in comparison to wild-type (LT_50_ of 4.8 h *vs* 6.3 h, respectively) (Figure 3C). Interestingly, in cholesterol-supplemented media, *cup-1(gk245)* animals also showed higher sensitivity to heat compared to wild-type, with a 14.6% thermoresistance reduction (LT_50_ of 6.4 h *vs* 7.5 h, respectively).

It is also known that cholesterol deficiency affects locomotion in *C. elegans* [12], [17]. Thus, we explored wild-type and *cup-1(gk245)* animals mobility using video recordings. In the case of *cup-1* mutant animals, we observed a reduction in worm speed regardless of cholesterol availability (Figure 3D). In cholesterol-supplemented media, the average speed of *cup-1(gk245*) animals was 0.105±0.036 mm/s *vs* 0.173±0.029 mm/s of wild-type, representing a 39% reduction in worm speed (Video S1). In the case of animals grown in low-cholesterol conditions a 36% reduction in worm speed was observed in *cup-1(gk245)* animals compared to wild-type: 0.066±0.009 mm/s *vs* 0.103±0.014 mm/s respectively (Video S2). In both cases, differences in speed were independent of food availability (data not shown and Video S3). Knockdown of *cup-1* recapitulated the mutant phenotype as RNAi animals showed a 40% decrease in speed in comparison to control animals (0.060±0.01 mm/s *vs* 0.100±0.01 mm/s, respectively) grown in low-cholesterol conditions (Figure S1 D). In all cases, subjects presented normal responses to mechanical stimuli, such as tap or touch (data not shown).

All phenotypes observed in *cup-1* defective animals, such as decrease in size, thermoresistance and locomotion, as well as impairment in larval development observed when grown in low-cholesterol conditions, suggest that the CUP-1 is a protein involved in cholesterol metabolism in *C. elegans*.

### CUP-1 defective animals show a decrease in cholesterol uptake

**Figure 4:**
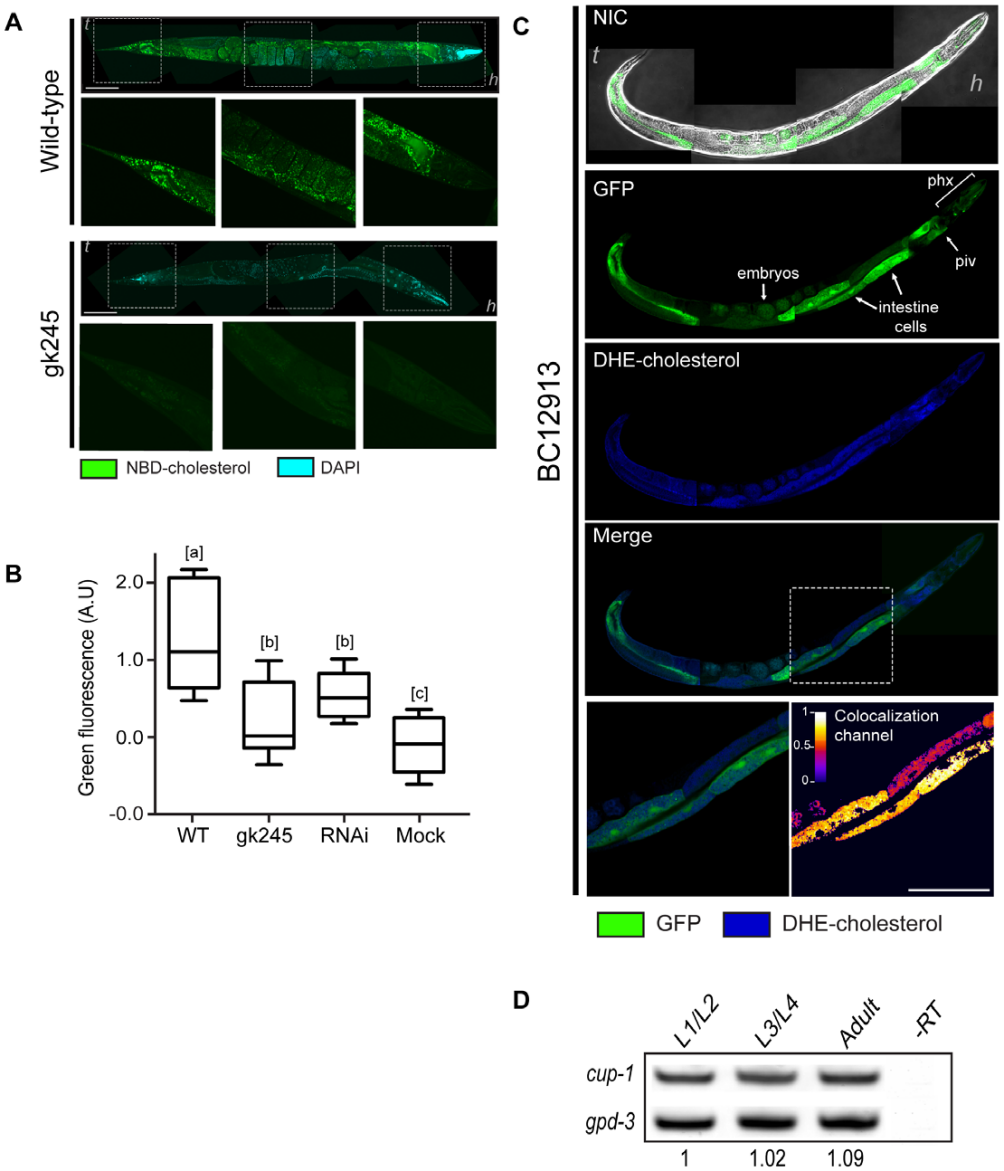
Impairment of cholesterol uptake in *cup-1* mutant animals and expression of *cup-1* in *C. elegans*. (A) Animals were grown in the presence of the cholesterol analog NBD and fluorescence was assessed by confocal microscopy. The upper panel shows a representative N2 wild-type animal. DAPI signal is shown in blue. Rectangles indicate zoom areas to the tail, mid section and head (only green channel). The lower panel shows a representative *cup-1(gk245)* animal. Scale bar: 100 µm. (B) Boxes represent first to third quartiles around the median of green channel intensity of wild-type animals (WT) fed with an empty plasmid in comparison to *cup-1(gk245)* and RNAi animals grown in the presence of NBD. Mock: basal autofluorescence from animals grown without NBD. The mean basal autoflurescence value of mock animals was taken as zero. Different letters [a], [b] and [c] indicate statistically significant differences per parameter among experimental groups, p<0.05. Bars: min. and max values; A.U: arbitrary units; n>20 for each condition. (C) The BC12913 strain (expressing GFP under *cup-1* promoter) was grown in the presence of the fluorescent cholesterol analog DHE and colocalization was assessed by confocal microscopy. Upper panels show Nomarski image overlapped with the green channel where GFP signal is observed in the pharynx, nerve ring and intestine cells (arrows). DHE signal is shown in blue. The rectangle indicates the zoom area of intestine cells presented in the lower left panel. A colocalization channel (lower right panel) was built from positive PDM values resulting from both pixels above the mean. *t*: tail; *h*: head; phx: pharynx; piv: pharyngeal-intestinal valve. Scale bar: 50 µm. (D) RT-PCR of *cup-1* transcript evaluated during larval development. GADPH (*gpd-3*) was used as control. No difference in *cup-1* expression was observed over time. Numbers indicate the rate cup-1/gpd-3. L1/L2 expression was taken as 1. −RT: negative control without reverse transcriptase.

After showing that *cup-1* defective animals were hypersensitive to cholesterol limitation, we explored if CUP-1 was involved in cholesterol uptake. To this purpose, *cup-1(gk245)* animals were grown in the presence of the green-fluorescent cholesterol analog NBD. A substantial decrease in NBD uptake was observed in *cup-1* mutant animals in comparison to wild-type animals that showed NBD-fluorescence in vesicle-like structures distributed in a punctuate pattern through the worm body including embryos (Figure 4A). These vesicle-like structures appeared to be intracellular and corresponded to previously reported cholesterol vesicles in *C. elegans* [18]. The green-NBD signal was especially notorious in cells of the digestive tract including the pharynx and the nerve ring, in agreement with previously reported observations using filipin staining [12]. Another cholesterol-NBD rich zone was observed in the tail. A complete 3D reconstruction of a wild-type animal grown with NBD cholesterol is presented in Video S4.

Densitometry analysis of the green channel intensity of animals grown in the presence of NBD, showed over 80% reduction in fluorescent signal in the *cup-1(gk245)* animals in comparison to wild-type (Figure 4B). Once more, knockdown of *cup-1* via RNAi recapitulated the mutant phenotype, as a 57% decrease in NBD-fluorescence was observed in *cup-1(RNAi)* animals in comparison to control.

Because NBD-cholesterol has a fluorescent molecule attached that could alter cholesterol properties, we decided to use radioactive cholesterol to corroborate our data. For this purpose, *cup-1(gk245)* mutant animals were grown in the presence of [^3^H]-cholesterol and accumulation of radioactivity was quantified in the progeny. Radioactive signal in CUP-1 mutant was reduced by 53% (Figure S3), probably as a result of a decreased cholesterol uptake in these animals. This result is consistent with our previous observations measuring NBD-cholesterol fluorescence.

*C. elegans* NCR-1 and NCR-2 proteins and their human homolog are involved in intracellular cholesterol trafficking [25], [27], [34], [35] but whether they are able to uptake cholesterol from the environment has not been yet observed. Therefore we decided to test the capability of *ncr-1(nr2022)* and *ncr-2(nr2023)* mutant animals to uptake radioactive cholesterol. *ncr-1(nr2022);ncr-2(nr2023)* double mutant animals presented a similar [^3^H]-cholesterol uptake compared to wild-type (Figure S3). To explore redundancy between *ncr* and *cup-1* genes, we made double mutant animals [*cup-1*(*gk245*);*ncr-1*(*nr2022*) and *cup-1(gk245);ncr-2(nr2023*)] and tested their cholesterol uptake capability. CUP-1 double mutant animals showed an equivalent reduction in [^3^H]-cholesterol incorporation as the *cup-1* single mutant (Figure S3). These data suggest that CUP-1 is important for cholesterol uptake from the environment, while NCR-1 and NCR-2 apparently are not required for this.

In support of these observations, growth of *ncr-1(nr2022);ncr-2(nr2023)* double mutant animals in the presence of NBD-cholesterol showed not significant alterations in fluorescent distribution (data not shown). This result is in agreement with recent observations showing that knock-down of *ncr-1* or *ncr-2* does not affect sterol distribution measured by DHE-cholesterol bleach-rate imaging [27]. Altogether, these observations support our hypothesis of CUP-1 as a novel protein involved in environmental cholesterol uptake in *C. elegans*.

### The expression pattern of CUP-1 correlates with cholesterol localization in *C. elegans*

To investigate the expression pattern of *cup-1* in *C. elegans*, we used the reporter strain BC12913 [36] that expresses the green fluorescent protein (GFP) under the *cup-1* promoter. The GFP signal was especially strong all along the worm intestine (Figure 4C). The pharynx also showed GFP signal, especially at the terminal bulb and presumably, the excretory gland cells. Although fluorescence was not as strong as that observed in other structures, GFP was also observed in embryos. The BC12913 reporter strain was also grown in the presence of the naturally occurring blue-fluorescent cholesterol analog DHE [37] and the colocalization pattern was evaluated by confocal microscopy. Analysis of the GFP and DHE fluorescence revealed a strong colocalization signal in the intestine cells (Figure 4C, lower panels). Taken together, these observations support the association between *cup-1* expression and cholesterol localization, particularly in the worm intestine.

To further investigate the timing of expression of *cup-1* in *C. elegans*, we performed RT-PCR in different developmental stages. Interestingly, *cup-1* expression was detected in all developmental stages and no differences were detected in mRNA levels (Figure 4D). This observation is especially significant as sterol uptake has been detected in all larval stages [18].

### Subcellular localization of CUP-1 in mammalian cells is compatible with endocytic pathways of cholesterol uptake

Due to the difficulty of using organelle markers directly in *C. elegans*, which possesses a highly impermeable body cuticle, and because no nematode cell-lines are available to this date, we explored CUP-1 subcellular localization taking advantage of mammalian cell lines. We generated a chimeric protein between *C. elegans* CUP-1 and the green fluorescent protein at the C-terminus, the construct was transfected in human embryonic kidney cells (HEK293 FT). The subcellular localization of this fusion protein, (CUP-1-GFP) was evaluated by confocal microscopy, and Manders' overlap coefficients (R) where calculated from colocalization channels [38].

**Figure 5:**
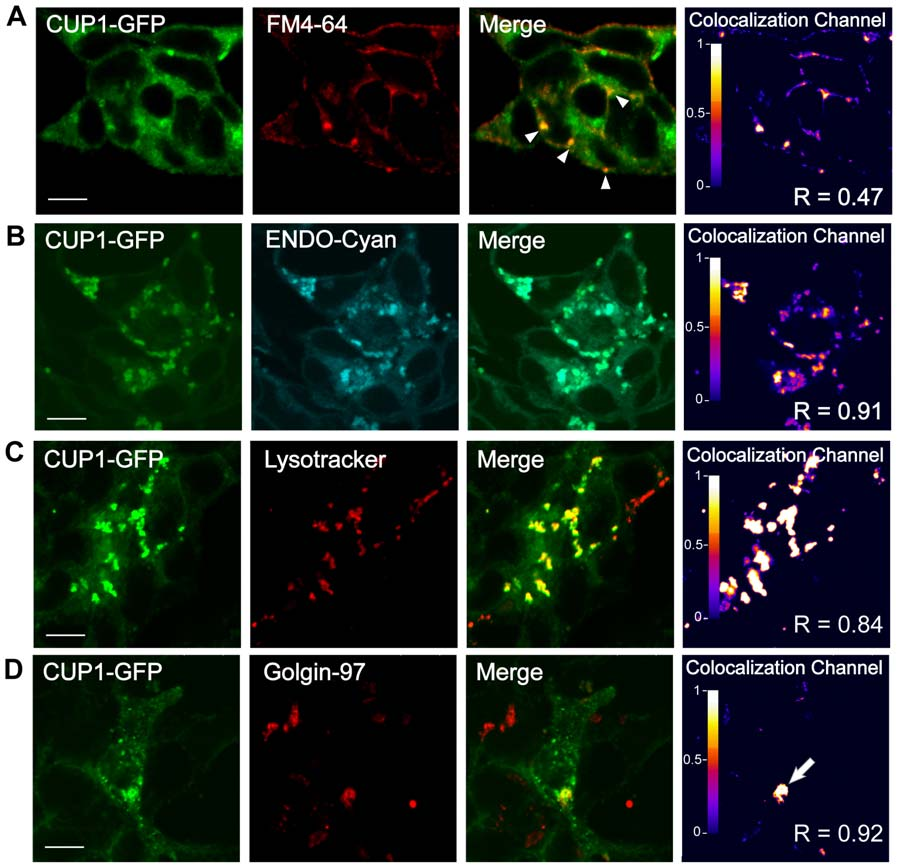
CUP-1-GFP expressed in mammalian cells presented a vesicle-like localization pattern. Chimeric protein was transiently expressed in HEK293 cells and colocalization with organelle cell markers was evaluated by confocal microscopy in living cells. Colocalization channel was built from positive PDM values resulting from both channels pixels that were above the mean. (A) Colocalization of CUP-1-GFP and the lipophilic membrane marker FM4-64 (red) was observed in membrane areas as well as endosome-like structures (arrow heads). These endosomes-like structures also colocalized with the endosome marker rho-GTPase in cyan (B) and within lysosomes by the use of red-Lysotracker (C). (D) Immunostaining with human Golgin-97 antibody (red) showed a positive colocalization signal at the Golgi (arrow). R: Manders' Overlap coefficient. Scale bar 10 µm.

In general, the CUP-1-GFP signal was detected in a punctuated pattern resembling the vesicle-like structures observed in *C. elegans* (compare Figure 5 green channel and Figure 4A). Remarkably, GFP signal colocalized with the plasma membrane marker FM4-64 (R = 0.47) (Figure 5A). Biotinylation of plasma membrane proteins also resulted in biotinylation of the CUP-1-GFP protein, corroborating the presence of the chimeric protein at the plasma membrane (data not shown). Colocalization signal was also observed in endocytic vesicles, as a result of FM4-64 endocytosis. These structures might correspond to early endosomes (see arrows in Figure 5A). To further investigate the nature of these vesicles, we used selective endosome and lysosome markers. We observed a strong colocalization signal between CUP-1-GFP and the endosome marker RhoB (R = 0.91) and Lysosotracker (R = 0.84) (Figure 5B and 5C, respectively) but not with mitochondrial or nuclear markers (data not shown). Additionally, inmmunostaining against the human Golgin-97 showed the presence of CUP-1-GFP in the Golgi (R = 0.92) (Figure 5D). All these colocalization studies of CUP-1-GFP support the possible involvement of CUP-1 in an intracellular cholesterol endocytic pathway.

### CUP-1 CRAC motifs directly interact with the cholesterol analog DHE

**Figure 6:**
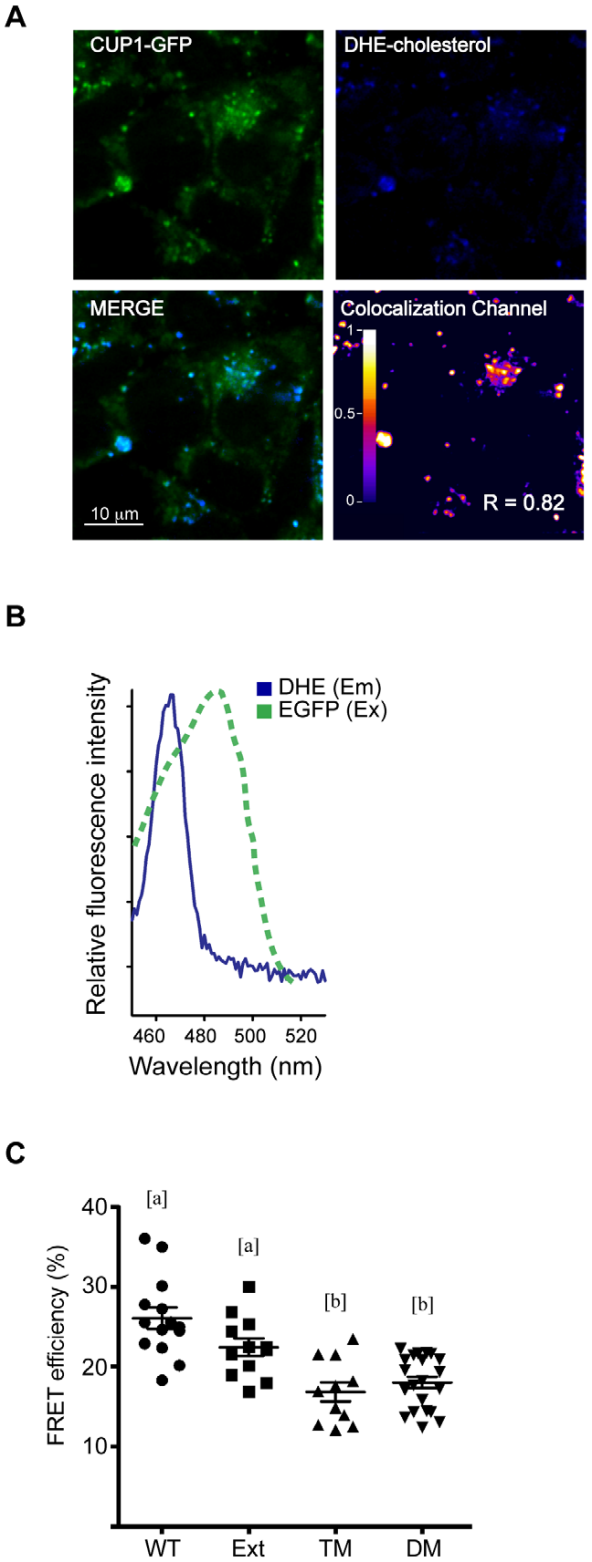
Interaction between CUP-1-GFP and the blue-fluorescent cholesterol analog DHE. HEK293 cells transiently expressing CUP-1-GFP were incubated with DHE cholesterol and fixed. (A) Subcellular localization was evaluated by confocal microscopy; colocalization channel was built from the positive PDM values from blue and green pixels that were above the mean. For clarity, pseudocolor RedFire table was used. R: Manders' Overlap coefficient. Scale bar 10 µm. (B) Spectral overlap between DHE emission (assessed by em-scan at 405 nm) and EGFP excitation spectra (Invitrogen Fluorescence SpectraViewer). (C) FRETeff was assessed after acceptor photo-bleaching in fixed cells expressing the wild-type (WT) CUP-1-GFP or mutagenized chimeric proteins. Ext: Mutagenized extracellular CRAC motif; TM: mutagenized transmembrane CRAC motif; DM: double mutant comprising Ext and TM mutations. Different letters [a] and [b] indicate statistically significant differences per parameter among experimental groups. Dot plot show the mean. p<0.05. Error bar: sem; n>12.

In order to determine if cholesterol colocalized with CUP-1, CUP-1-GFP-transfected mammalian cells were incubated with the blue-fluorescent cholesterol analog DHE and colocalization signal was evaluated. A positive colocalization signal was observed with a Manders' overlap coefficient of 0.82 (Figure 6A). Although colocalization and the estimation of Manders' coefficients are good ways to evaluate vicinity between molecules, a different approach is required to confirm a molecular interaction. Thus, we took advantage of the spectral overlap between the emission peak of DHE at 467 nm and the excitation peak of GFP at 488 nm (Figure 6B) to measure the Förster Resonance Energy Transfer (FRET) between these two molecules. We observed a robust energy transfer between CUP-1-GFP and DHE with over 28% of FRET efficiency (FRETeff), indicative of a direct interaction between CUP-1 and the cholesterol analog (Figure 6C). Importantly, using soluble GFP as control resulted in zero FRETeff, demonstrating the specific interaction between CUP-1 and cholesterol.

To evaluate if the predicted CRAC motifs in CUP-1 are responsible for the direct interaction with cholesterol, we generated single mutants substituting tyrosine 126 and 576 (extracellular and transmembrane CRAC motifs, respectively) for the amino acid glycine. Additionally, a double mutant including both changes was generated. It has previously been demonstrated that mutation of this residue in CRAC motifs is sufficient to block the interaction with cholesterol [39]. Mutagenization of the transmembrane CRAC motif resulted in 16.7% of FRETeff, which represents a 41% reduction in comparison to wild-type CUP-1 (Figure 6C). Mutagenization of the extracellular CRAC motif did not result in a statistically significant reduction in FRETeff. This is expected as this CRAC motif and the GFP are in opposite sides of the membrane, making FRET impossible between these fluorophores. In the case of the double mutant, FRETeff was equivalent to that of the single transmembrane CRAC mutant (17.8% in FRETeff), corroborating that the extracellular CRAC motif was not contributing to FRET. Taken together, these data confirm the direct interaction between cholesterol and the transmembrane CRAC motif of CUP-1.

## Discussion

In the present work, we have collected evidence supporting the role of Cholesterol Uptake Protein-1 (CUP-1), formerly known as *tag-130*, as a membrane-associated protein involved in the uptake of dietary cholesterol in *C. elegans*. Silencing of *cup-1*, by RNAi or its absence in the *cup-1(gk245)* mutant, resulted in animals sensitive to cholesterol availability. In low-cholesterol conditions, we observed subnormal animal size (over 60%), and decreased fertility (over 70%) associated to reduced oocyte number, increased embryo lethality, impairment in larval development and alterations in oocyte distribution inside the gonad. All these phenotypes may be a consequence of a reduction in steroid hormones due to the lack of cholesterol. Another possible explanation might be the failure of cholesterol to attach to morphogen molecules (e.g. hedgehog-like), acting between somatic gonad cells and oocytes, as previously suggested by several authors studying sterol starvation in *C. elegans* [2], [6], [40].

The wild-type strain also showed a reduction in animal size and offspring number growing in low-cholesterol conditions; however, it was not as dramatic as in the *cup-1* mutant animals. Additionally, wild-type animals did not show the alterations in oocyte morphology seen in *cup-1* mutant animals, suggesting a higher sensitivity of the CUP-1 defective animals to cholesterol availability. Remarkably, sperm cell number was unaffected in *cup-1(gk245)* animals in agreement with the notion that cholesterol is not essential for spermatogenesis [18].

We also observed a higher sensitivity to heat stress in *cup-1* mutant animals growing in low-cholesterol conditions, suggesting impairment of stress resistance in these animals. However, staining of lipids with Sudan Black, Oil Red-O or Nile Red did not reveal any alteration in distribution or quantity of lipid storages in *cup-1(gk245)* animals (data not shown), suggesting that in this case, the lower thermoresistance was related to low cholesterol levels. Interestingly, although the lack of cholesterol had a clear effect in locomotion in the *cup-1* mutant or RNAi animals, in standard cholesterol supplementation subjects were also slower than the control. This interesting observation deserves further investigation.

Evaluation of cholesterol uptake capacity in animals lacking CUP-1 showed a decrease in signal of radioactive cholesterol and NBD fluorescent analog. These observations suggest the involvement of CUP-1 in *C. elegans* cholesterol retention and/or uptake. The absence of two other genes involved in cholesterol transport, *ncr-1* and *ncr-2*, did not have a significant effect in cholesterol uptake, which is in agreement with recent studies showing that these proteins are implicated in intracellular release of cholesterol from endocytic structures and not in the initial uptake from the environment [27]

*In vivo* DHE labeling in the GFP-reporter strain showed that cholesterol accumulation correlated with CUP-1 expression mainly in the digestive tract cells, the intestine is the main site of lipid storage and modification as well as the tissue with initial contact with absorbed sterols [1], [12], [18]. These data support the notion that the main site for cholesterol entrance in *C. elegans* is the gut and not the cuticle. Expression of *cup-1* also correlated with cholesterol accumulation in the pharyngeal intestine valve and the terminal bulb of the pharynx, that may include the nerve ring, a structure where cholesterol plays an essential role in dauer induction and sensory processing (e.g. chemotaxis, thermotaxis) [16], [26]. *Cup-1* expression was also observed in a structure that possibly correlates with the excretory gland cells, the initial place for storage of unmodified ingested-sterols [41]. Signal in the tail region suggests zones of late-sterol metabolite accumulation, such as the phasmid socket cells [12], [18]. In addition, we observed *cup-1* expression and cholesterol colocalization in embryos, where CUP-1 may be playing a redundant role with the vitellogenin-mediated endocytosis of lipoproteins. This redundancy may reflect the fundamental role of cholesterol during fertilization and embryo development.

It is well known that cholesterol uptake starts after hatching and continues throughout the worm's life. In contrast to vitellogenins [18], we detected *cup-1* expression in all developmental stages, making CUP-1 a good candidate to explain early cholesterol uptake.

Although CUP-1 could be responsible for an important fraction of the whole cholesterol uptake in *C. elegans*, it is clear that additional pathways may exist, given the fact that CUP-1 defective animals showed normal size and offspring in the presence of cholesterol and ultimately by the fact that the absence of CUP-1 was not lethal. It has been established that a small quantity of cholesterol in the media supports reproduction and allows the production of animals of normal size [12]. It is only when CUP-1 defective animals are grown in low cholesterol conditions that the phenotype associated with lack of CUP-1 becomes apparent. In this regard, basal endocytosis in the intestine could satisfy sterol needs. This effect is evident by quantifying the radioactive cholesterol signal where about the half of the basal incorporation of cholesterol is still observed in CUP-1 mutants.

Mutagenization of the conserved transmembrane CRAC motif of CUP-1 resulted in a 41.2% decrease in FRETeff between the mutant protein and the blue-fluorescent cholesterol analog DHE. This result not only indicates proximity between CUP-1 and cholesterol in the cholesterol-rich vesicles, it also suggests direct interaction between molecules. Transmembrane CRAC motifs have been identified as a cholesterol-binding site in proteins capable of mediating cholesterol transport across membranes such as the human Peripheral-Type Benzodiazepine Receptor [39].

Mutagenization of the extracellular CRAC motif did not have a substantial impact in FRETeff. This result was expected as the minimal distance for FRET is 10 nm [42], and in this case, the two fluorophores involved in FRET are in opposite sides of the membrane making impossible for FRET to occur. In this regard, no additive effect was observed in the CRAC double mutant (that was equivalent to the transmembrane CRAC mutant), corroborating the interaction between cholesterol and the transmembrane motif. However, we cannot discard the involvement of the extracellular CRAC motif in cholesterol interaction/recognition, as extracellular cholesterol-binding sites have been suggested to act as initial recognition site for sterols in proteins involved in cholesterol uptake in mammals [43]. Further experiments will be needed to address the functionality of the extracellular CRAC motif in CUP-1 by positioning GFP in an extracellular domain.

Although we observed a significant reduction in FRETeff, residual energy transfer was evident in the transmembrane CRAC mutant. At least two non-exclusive explanations could account for this observation: 1) basal interactions with membrane-cholesterol could occur in the cholesterol-rich vesicles, and 2) other uncharacterized motifs in CUP-1 could be involved in cholesterol interaction. It would be interesting to explore if the complete deletion of the CRAC motif, instead of a single amino acid change, could have a larger effect in FRETeff.

The CUP-1-GFP fusion allowed us to identify the subcellular localization of CUP-1 in mammalian cells. We detected its presence in the plasma membrane as well as within endocytic structures. Interestingly, we also detected CUP-1-GFP in lysosomes. This observation correlates with the identification of two lysosome-targeting signals in the first intracellular loop of CUP-1 (Figure 1).

Overall, the evidence collected so far lead us to propose that CUP-1 could be involved in dietary-cholesterol uptake from the environment in *C. elegans*. At the molecular level, CUP-1 could be participating in cholesterol recognition at the plasma membrane of intestinal cells, e.g. by the extracellular CRAC motif. In a subsequent step, cholesterol interaction with the transmembrane CRAC motif might produce a conformational change inducing its internalization in a similar manner to that described for hNPC1L1 [43], the protein responsible for intestinal sterol absorption in humans. Indeed, in an additional analysis we have identified two extracellular and two transmembrane CRAC motifs in hNPC1L1 (amino acids: 473–480, 558–567 and 633–641, 700–705, respectively). In a later step, cholesterol could scape from endosomes/lysosomes to the ER and Golgi. The presence of CUP-1-GFP in endosomes, as well as in lysosomes and in Golgi is compatible with this hypothesis. In mammals, endocytosis of cholesterol and its posterior release from the endocytic structures to the ER and Golgi are the first crucial steps in sterol absorption and metabolism [44], [45]. Still, a parallel role of CUP-1 in intracellular cholesterol metabolism cannot be discarded.

We tested the ability of mammalian cells expressing CUP-1 to internalize fluorescent cholesterol analogs; however, we failed to detect an increase in cholesterol uptake measured by flow cytometry (data not shown). In this regard, mammalian cells present a strong constitutive cholesterol uptake that could mask CUP-1-mediated uptake. Nevertheless, it has been previously reported that the knockdown of one of the putative CUP-1 human homologue genes (hSIDt1), resulted in the inability of the cells to internalize siRNAs only when they are covalently attached to cholesterol [46], [47]. This is a very relevant result as it proves that the human homolog of CUP-1 in fact has a role in cholesterol uptake.

It has been suggested that hSIDt1 and hSIDt2 are human homologues to *C. elegans* SID-1 (the protein responsible of the systemic RNAi effect in nematodes, as it mediates the entrance of dsRNA into cells) [48], [49]. Nevertheless, we and others have observed that the lack of CUP-1 does not affect the systemic RNAi pathway of *C. elegans* (data not shown and [50]). Moreover, a phylogenetic analysis of CUP-1 homologues from different species showed that the mammalian proteins SIDt1 and SIDt2 have a higher identity to CUP-1 than to SID-1 (on average, 31.5% *vs* 16.4% identity, respectively) suggesting that mammalian SIDt1 and SIDt2 are homologs of CUP-1 and not of SID-1 (Figure S4 A). More importantly, we identified CRAC motifs in mammalian SIDt1 and SIDt2 proteins, but not in *C. elegans* SID-1 (Figure S4 B). If the involvement of *cup-1* mammalian homologue genes in cholesterol uptake is demonstrated, it may prove the existence of a new family of proteins involved in cholesterol metabolism.

## Materials and Methods

### Ethics Statement

N/A.

### Materials

22-(N-(7-nitrobenz-2-oxa-1,3-diazol-4-yl)amino)-23,24-bisnor-5-cholen-3-ol (NBD), DAPI, organelle cell markers, antibody, Opti-MEM, Dulbecco's medium (DMEM) and enzymes were purchased from Invitrogen (Carlsbad, CA. USA). Dehydroergosterol (DHE), cholesterol, molecular biology grade ethanol, glycine, IPTG, antibiotics and Salts from Sigma-Aldrich (St. Louis, MO. USA). [1,2-^3^H]-cholesterol (50 Ci/mmol) was purchased from PerkinElmer (Boston, MA, USA). Bactotryptone (pancreatic digest) and Agar were from BD (Sparks, MD. USA). NaCl and Potassium phosphate from J.T.Baker (Phillipsburg, NJ. USA). Granular paraformaldehyde (PFA), 25 mm circular cover glass and 9 mm coversilps (.13–.17 mm) were products of Electron Microscopy Sciences (Washington, PA. USA) Fluorescent Mounting Media (FMM) was from DakoCytomation (Denmark). CUP-1 topology model was generated in the TMHMM Server v.2.0 [51]. **Animals and Media**. N2 variety Bristol was used as wild-type. The *cup-1(gk245)* mutant (VC452) and *ncr-1(nr2022);ncr-2(nr2023)* (JT10800) strains were obtained from the CGC (U of Minesota). Double mutant animals were obtained by mating *cup-1gk(245)* males with *ncr-1(nr2022);ncr-2(nr2023)* hermaphrodites. To corroborate mutants alleles in the offspring, the following primers were used: CAGCAAAGATCTGCACCGTA and CGTAAAAGCTTGACATGCGA for *gk(245)*; TCTCTTCTTGTCGAGCCCTGG and CAGAGTACCAACCAGCTGAGGC for *nr2022*; and GTGTGGTGAGTGGTGTAGACTAGGAC and CAGCAGTATGAATTTCTTCCTGG for *nr2023*. GFP reporter strain BC12913 (carrying sIs10486 transgene) was kindly provided by the BC *C. elegans* Gene Expression Consortium. All strains were propagated at 20°C according to standard procedures in nematode growth medium (NGM) with 2% agar and supplemented with 8 µg/ml of cholesterol (added from stock solution 20 mg/ml in MB grade ethanol). Overnight cultures of *E. coli* OP50-1 were washed three times in Milli-Q water before seed as food source. For the low-cholesterol condition, cholesterol supplementation was omitted and ethanol was added instead.

### Worm Cholesterol Sensitivity Assays

As maternally derived sterols can supply cholesterol needs in the first generation of worms [12], all the experiments were performed in F2 progeny growing in low-cholesterol NGM plates (20°C). For fertility assays a single L4 hermaphrodite was transferred to a 35 mm NGM plate and moved to new plate every 24 h. Sums of all larvae from 5–6 days are shown from three independent experiments each one with more than 20 worms. Gonads were dissected, fixed and mounted after standard DAPI staining and observed in a Nikon E600 epifluorescence microscope with 40× objective and AxioCam-MRc camera (Zeiss). For size quantification: four L4 hermaphrodites were transferred to 60 mm NGM plates and progeny samples were daily collected and fixed. Images were taken in an Olympus FV-10 Confocal Microscope with 10× objective and worm area was measured with FluoView software V2.1 (Olympus Co. Japan). An average of over 25 worms for each day is presented from two independent experiments. To evaluate embryonic lethality, L4 hermaphrodites were individually transferred to NGM plates. After 24 h embryonic lethality was calculated as the number of non-hatched embryos divided by the sum of all embryos. The percent of development was calculated from the number of hatched embryos that reach adulthood. An average from three independent experiments is presented. For dauer analysis, 12–15 adults were seeded in 60 mm NGM plates and maintained at 25°C for 14–15 days. Animals were collected, counted and 2000 worms were treated with 1% SDS for 30 min and then seeded in NGM plates; after 2 h of recovery the animals that survived were counted as dauers [52]. For velocity measures, an area of 12 cm^2^ of a 60 mm NGM plate was delimitated with Whatman paper No. 1 soaked in 50 mM CuCl_2_. Twenty worms were transferred and 8–10 min of video were recorder (320×240, 3 fps) in a Nikon stereoscopic microscope with an adapted digital camera. Individual worms were automatic identified with the Parallele WormTracker [53] using the following settings: worm area 6 to 40 pixels; Max Distance: 20 pixels; Max Size Change: 25 pixels; Shortest valid track 70 frames. Then, velocity was calculated with WormAnalyzer software [53] calibrated to 17.5 pixels/mm. Average speed measures are presented from over 20 recordings from three independent experiments. For thermoresistance experiments, one day-old adults worms were transfer to 35 mm NGM plates and incubated at 35°C for 1 h; viability was evaluated by touch stimuli before the next incubation at 35°C until all subjects were dead. At least 20 worms were analyzed by triplicate for each condition. Averages from three independent experiments were presented. In all cases, statistic analysis was performed by parametric t-test.

### *In vivo* fluorescent cholesterol labeling

Fluorescent cholesterol analogs were added to previously washed bacteria (OP50-1 or HB115 plus antibiotics) before seeding in 35 mm NGM plates. Final concentrations were 30 µM of NBD or 10 µM of DHE (from a fresh 10,000× stock solutions in ethanol). After overnight incubation at room temperature, plates were stored protected from light at 4°C for up to one week. One L4 hermaphrodite was transferred to plates and after 96 h at 20°C animals were collected and washed with M9 before fixing with 3% PFA in M9 for 30 min at room temperature. Fixed animals were washed and quenched with glycine 100 mM in M9 before being mounted on slides with FMM. Fluorescence was evaluated with a Spectral Laser Scanning (SLS) Olympus FV-1000 Confocal Microscope (40× N.A 1.3 oil immersion) using GFP settings for NBD and DAPI settings for DHE (sequentially acquired) with the FV10-ASW 2.1 software. The Multi area time laps option was used for 3D reconstruction of complete worm. For densitometry analysis, images were taken with an AxioCam-MRc camera (Zeiss) in a Nikon E600 epifluorecence microscope with Endow GFP HYQ Filter Cube (ex 450/490, 495DM, em 500/550) and 10× objective. ImageJ was used for quantification of the green channel [54] of each nematode after subtracting background signal from the same image. Average of over 20 subjects is presented. Differences were evaluated by one-way ANOVA followed by Bonferroni's Multiple Comparison Test. Differences were indicated by different letters (a>b>c) where p<0.05 was considered statistically significant.

### Radioactive cholesterol uptake

[^3^H]-cholesterol was added to washed bacteria (250 nM) that were seed on 60 mm NGM plates. After overnight incubation at room temperature, 4 adult animals were transferred to these plates and incubated at 20°C for four days. Adult progeny was collected in groups of 10 and lysed in worm-lysis-buffer plus Proteinase K. Uptake of ^3^H-cholesterol was quantified by liquid scintillation spectrometry. Differences were evaluated by one-way ANOVA followed by Bonferroni's Multiple Comparison Test.

### RNAi feeding

*cup-1* was amplified from a *C. elegans* cDNA library using the primers ATGAGGACCTCACAGGCG and CTAGAAAACTCGAATTGTATTCC. A HindIII/HindII DNA fragment (530 bp) was cloned into the feeding vector pPD129.36 and transformed into *E. coli* strain HT115. The overnight culture (50 mg/ml ampicillin, 12.5 mg/ml tetracycline) was washed three times with Milli-Q water and induction was performed by overnight incubation at room temperature on 60 mm NGM plates containing antibiotics and 1 mM of IPTG as in [55]. To evaluate cholesterol uptake, NBD was added to washed bacteria before seeding (30 µM). Induced plates were stored at 4°C for up to one week. Four L4 hermaphrodites were transfer to NGM plates and after 24 h at 20°C adults were removed. Adult F1 worms were collected with M9 and fixed with 3% PFA in M9 (60 min at 4°C). After background quenching with 100 mM glycine in PBS, animals were mounted with FMM. Knockdown efficiency was corroborated by real-time RT-PCR using the following primers: ATGAGGACCTCACAGGCGATT and GTACACCACATCCCATTTCGCT. The empty pPD129.36 plasmid was used as control.

### CUP-1-GFP expression in mammalian cells

CUP-1 stop codon was removed by PCR using the following primers: TCTAGAATGAGGACCTCACAGGCG and GGATCCCCTCCGAAAACTCGAATTGTATTCC. The product was cloned in pEGFP-N1 from Clontech (Mountain View, CA. USA) and the chimeric vector was transfected in HEK293-FT cells using Lipofectamine/PLUS Reagent (Invitrogen) in 35 mm dishes according to manufacture instruction. After 24 h cells were trypsinized and seed on circular coverslips. Cells were maintained in DMEM (with glutamine and pyruvate), supplemented with 10% of heat inactivated Fetal Bovine Serum (Wisent, premium quality. Canada) at 37°C and 5% of CO_2_ for 24–36 h. FM4-64 and Lysotraker were incubated in Opti-MEM according to manufacturer instructions. *In vivo* images were sequentially acquired using a SLS Olympus FV-1000 Confocal Microscope (60× N.A. 1.45 oil immersion, zoom 5, Kalman filter 2) equipped with a cell incubation system (INU-ZIL-F1, TOKAI HIT, Japan) at 37°C in Opti-MEM. Images were analyzed with ImageJ [54] using the Image Correlation Analysis plugin [56] from the WICF bundle [57]: background was subtracted independently to each channel and the green channel was used as region of interest (ROI). Resulted positive PDM (Product of the Differences from the Mean) images were pseudocolor in RedFire Lut. Manders' overlap coefficients where calculated from these colocalization channels from GFP positive cells using ImageJ Manders' Coefficients plugin [38], [56]. Immunostaining was performed in fixed cells using Anti-Golgin-97 antibody according to manufacturer instructions.

### FRET assay

CUP-1-GFP transfected cells were grown on micro coverslips and incubated with 5 µM DHE in Opti-MEM without FBS for 35 min at 37°C, 5% CO_2_. Cells were fixed with filtered 3% PFA in PBS for 20 min at room temperature. After extensive PBS washes and 10 min incubation in glycine 50 mM in PBS, coverslips were mounted with FMM and sealed with transparent nail polish. For FRET efficiency quantification in absolute terms, acceptor fluorophore (GFP) was selectively photobleached and the dequenching of the donor (DHE) fluorescence was measured [58]. Briefly, a circular ROI of about 50% of visible cell cytoplasm was selected and bleached using the *Tornado* function (40%, 488 nm; 1.5 s) in a SLS Olympus FV-1000 Confocal Microscope (60× 1.45NA oil immersion). Three consecutive images were taken before and after acceptor photobleaching of GFP and basal decay of fluorophores was assessed from the not-photobleached area of the same cell. FRET Efficiency is given by: FRETeff = (Dpost−Dpre)/Dpost (D = donor intensity) [42] calculated in FV-10 ASW 2.1 Software (Olympus, Japan). CUP-1-GFP CRAC single aminoacid changes were generated using QuikChange® Site-Directed Mutagenesis Kit (Stratagene, Santa Clara, CA. USA) according to manufacturer's instructions using the following primers: CACGTCCAGTGCATGGCAATTTCCGTGCAG and CTGCACGGAAATTGCCATGCACTGGACGTG for the extracellular motif and GTCAGCCTCGAGTTCGGTTTCAAAGGAATCTGG and CCAGATTCCTTTGAAACCGAACTCGAGGCTGAC for the transmembrane motif. All constructs were fully sequenced before transfection. Differences were evaluated by one-way ANOVA followed by Bonferroni's Multiple Comparison Test. Differences were indicated by different letters (a>b) where p<0.05 was considered statistically significant.

## Supporting Information

Figure S1 **Knockdown of** ***cup-1*** **resulted in oocyte alterations in subjects grown in low-cholesterol conditions.** (A) No alterations in spermatogenesis were observed in DAPI staining of wild-type and *cup-1(gk245)* spermatechaes from animals grown in standard cholesterol supplementation (upper panel) or in low-cholesterol conditions (lower panel). Scale bar 50 µm. (B) Relative expression (2∧-(ΔΔCt)) assessed by real time RT-PCR of *cup-1* in RNAi animals normalized to subjects fed with an empty plasmid (E.P) showed a ∼60% decrease in *cup-1* expression in RNAi animals. Error bars: sd. * p<0.001. (C) F1 control animals fed with an empty plasmid (E.P) or *cup-1(RNAi)* animals were grown with cholesterol supplementation (upper panel) or in low-cholesterol conditions (lower panel). For clarity, membranes of oocytes where a double-line array was observed are highlighted in white. Oocyte number was estimated form Nomarski images of F2 worms. + cholesterol: n>36; − cholesterol: n>53. (D) Speed was severely affected in *cup-1(RNAi)* animals. Worm speed was estimated form video recordings in plates without food of *cup-1(RNAi)* and control animals (fed with an empty plasmid) grown in low-cholesterol conditions. Box shows first to third quartiles around the median. Bars: min. and max. values. n>300 tracks; * p<0.0001.
(TIF)

Figure S2 **Normal development of wild-type and** ***cup-1*** **mutant animals grown in low-cholesterol conditions.** Nomarski images of wild-type and *cup-1(gk245)* animals grown with cholesterol supplementation or in low-cholesterol conditions. (A) Embryos from 2-cells to pretzel stage. Scale bar: 20 µm. (B) Larval development from L1 to adult; rectangles indicate the vulva. Scale bar: 50 µm.
(TIF)

Figure S3 **[^3^H]-Cholesterol Uptake is diminished in** ***cup-1*** **mutant animals.** Single and double mutant animals were grown in the presence of radioactive cholesterol and counts per minute (c.p.m) were assessed in the adult progeny. N2: wild-type; gk245: *cup-1(gk245)*; ncr-1: *ncr-1(nr2022)*; ncr-2: *ncr-2(nr2023)*. * p<0.01. Error bar: sem; n>300.
(TIF)

Figure S4 **Phylogenetic analysis of CUP-1 homologue genes.** Protein sequences of *C. elegans* CUP-1 homologues were aligned by ClustalW (MEGA5 [59]). (A) The multiple sequence alignment was then used to generate a phylogenetic tree by a maximum likelihood method using a WAG substitution model. Percentage of amino acid identity *vs C. elegans* SID-1 and CUP-1 are presented on the right. Numbers in branches indicate bootstrap values. (B) Alignment of the conserved extracellular and transmembrane CRAC motifs (L/V-X_(1–5)_-Y-X_(1–5)_-R/K) in CUP-1 homologue proteins. Numbers indicate amino acid position in CUP-1. m: *Mus musculus*; r: *Rattus norvegicus*; h: *Homo sapiens*; ce: *Caenorhabditis elegans*.
(TIF)

Video S1 Recordings of wild-type (N2) and *cup-1* mutant animals (*gk245*) grown in the presence of cholesterol. Speed was increased five times for clarity. Video was recorded without food.
(MOV)

Video S2 Recordings of wild-type (N2) and *cup-1* mutant animals (*gk245*) grown in low-cholesterol conditions. Speed was increased five times for clarity. Video was recorded without food.
(MOV)

Video S3 Recordings of wild-type (N2) and *cup-1* mutant animals (*gk245*) grown in low-cholesterol conditions. Speed was increased ten times for clarity. Video was recorded in the presence of food.
(MOV)

Video S4 3D reconstruction of a fixed adult wild-type hermaphrodite (N2) grown in the presence of the green cholesterol analog NBD. Images were taken by confocal microscopy with 10 µm Z-step.
(MOV)
