# Correction: CUP-1 Is a Novel Protein Involved in Dietary Cholesterol Uptake in *Caenorhabditis elegans*

**DOI:** 10.1371/annotation/5a203055-6c15-43b0-96ad-0fbd5eb9b810

**Published:** 2012-08-21

**Authors:** Victor J. Valdes, Alejandro Athie, Laura S. Salinas, Rosa E. Navarro, Luis Vaca

The authors have changed the name of the protein described in the paper from CUP-1 to ChUP-1, given that CUP-1 was already taken by a family of genes with unknown function. 

