# Supplementary figures and images for: CUP-1 Is a Novel Protein Involved in Dietary Cholesterol Uptake in Caenorhabditis elegans
Source: PLoS One. 2012 Mar 27;7(3):e33962. doi: 10.1371/journal.pone.0033962 (PMC3313951; doi:10.1371/journal.pone.0033962)

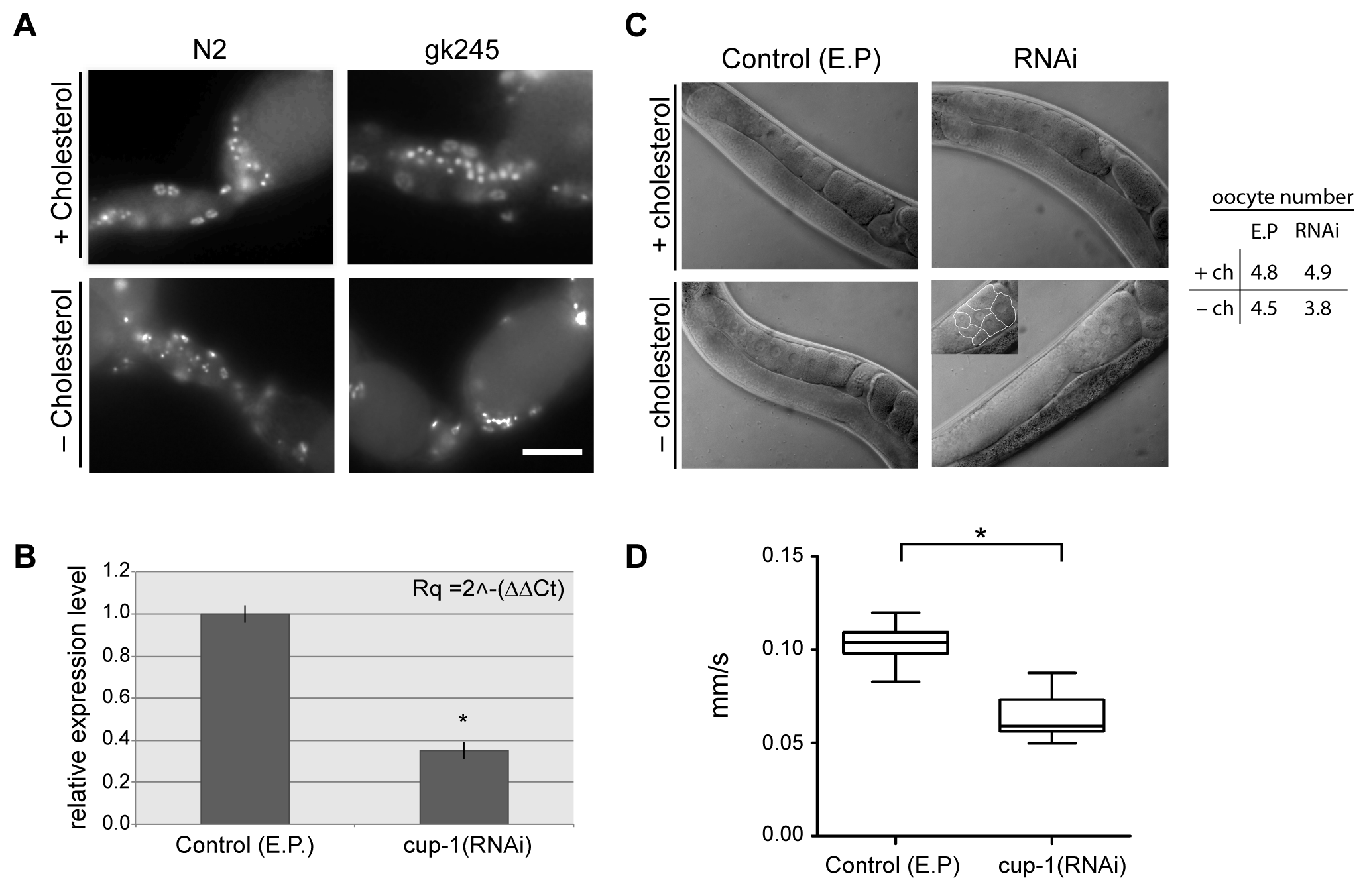

Supplement: Figure S1 — Knockdown of cup-1 resulted in oocyte alterations in subjects grown in low-cholesterol conditions. (A) No alterations in spermatogenesis were observed in DAPI staining of wild-type and cup-1(gk245) spermatechaes from animals grown in standard cholesterol supplementation (upper panel) or in low-cholesterol conditions (lower panel). Scale bar 50 µm. (B) Relative expression (2∧-(ΔΔCt)) assessed by real time RT-PCR of cup-1 in RNAi animals normalized to subjects fed with an empty plasmid (E.P) showed a ∼60% decrease in cup-1 expression in RNAi animals. Error bars: sd. * p<0.001. (C) F1 control animals fed with an empty plasmid (E.P) or cup-1(RNAi) animals were grown with cholesterol supplementation (upper panel) or in low-cholesterol conditions (lower panel). For clarity, membranes of oocytes where a double-line array was observed are highlighted in white. Oocyte number was estimated form Nomarski images of F2 worms. + cholesterol: n>36; − cholesterol: n>53. (D) Speed was severely affected in cup-1(RNAi) animals. Worm speed was estimated form video recordings in plates without food of cup-1(RNAi) and control animals (fed with an empty plasmid) grown in low-cholesterol conditions. Box shows first to third quartiles around the median. Bars: min. and max. values. n>300 tracks; * p<0.0001. (TIF) [file pone.0033962.s001.tif]

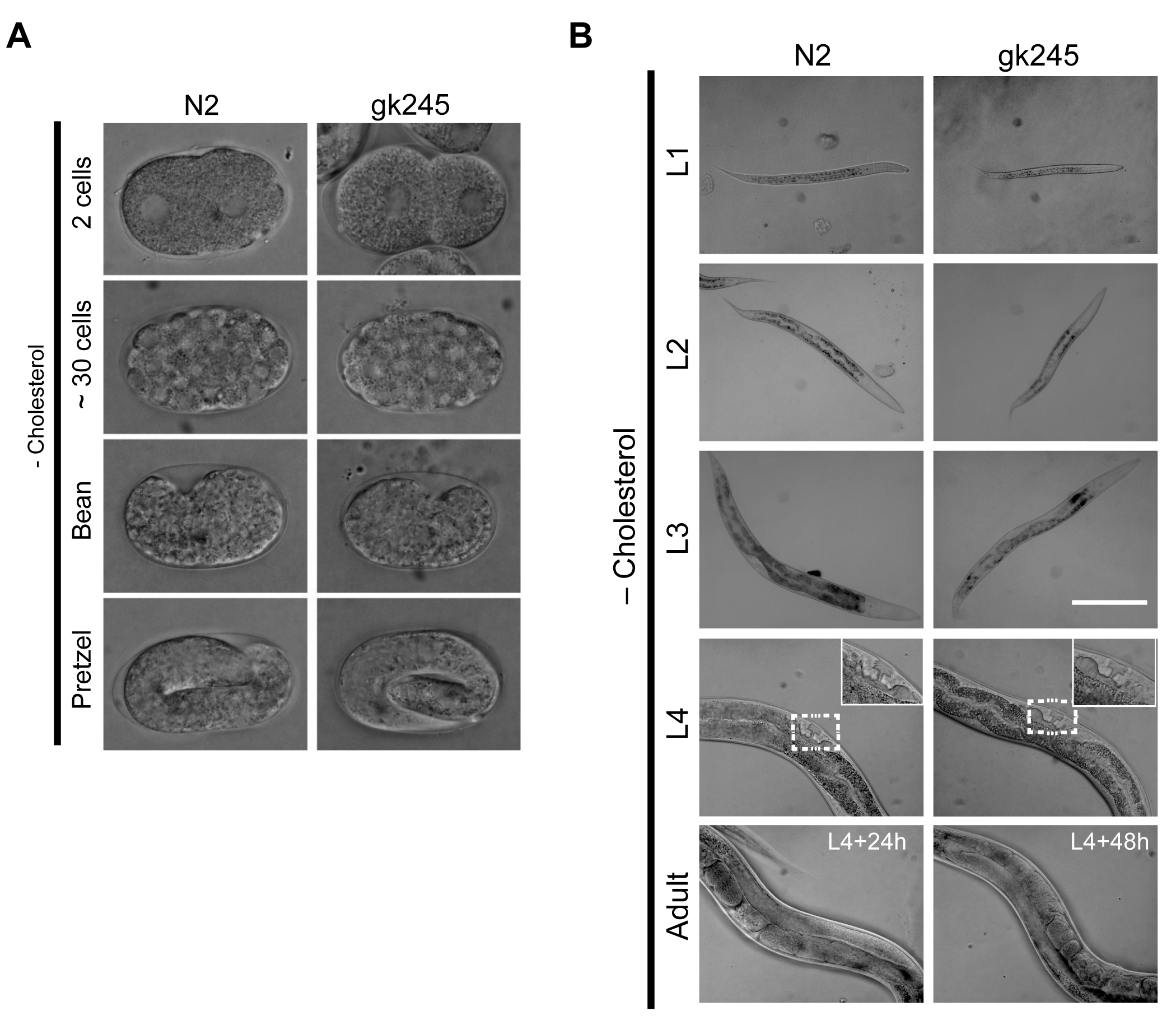

Supplement: Figure S2 — Normal development of wild-type and cup-1 mutant animals grown in low-cholesterol conditions. Nomarski images of wild-type and cup-1(gk245) animals grown with cholesterol supplementation or in low-cholesterol conditions. (A) Embryos from 2-cells to pretzel stage. Scale bar: 20 µm. (B) Larval development from L1 to adult; rectangles indicate the vulva. Scale bar: 50 µm. (TIF) [file pone.0033962.s002.tif]

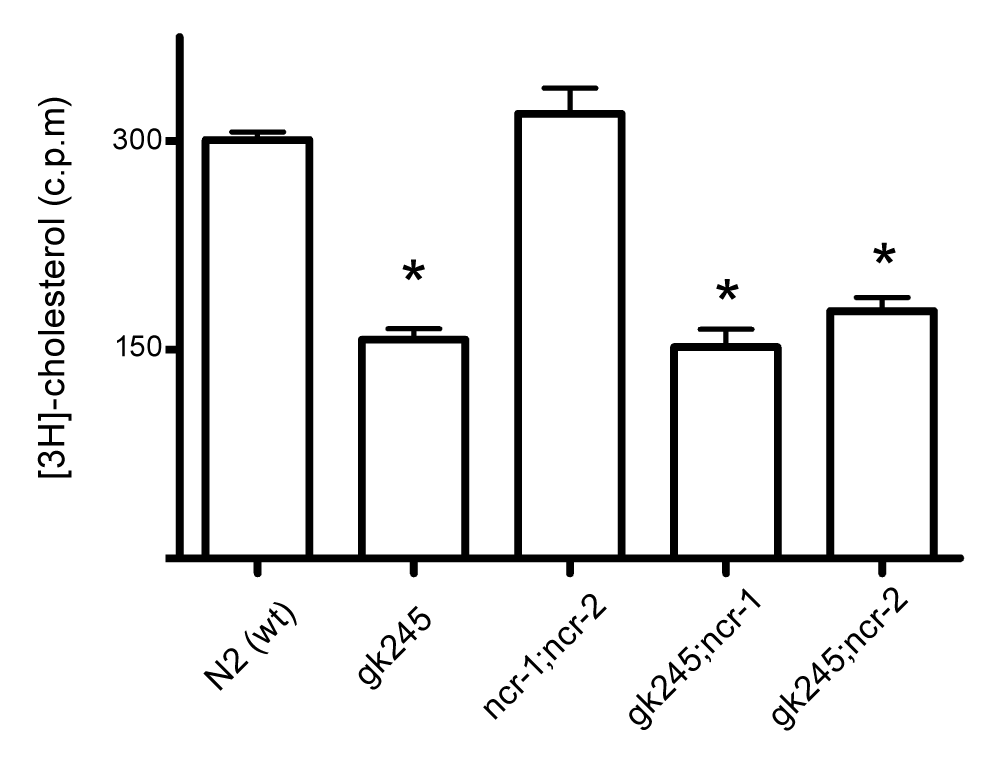

Supplement: Figure S3 — [3H]-Cholesterol Uptake is diminished in cup-1 mutant animals. Single and double mutant animals were grown in the presence of radioactive cholesterol and counts per minute (c.p.m) were assessed in the adult progeny. N2: wild-type; gk245: cup-1(gk245); ncr-1: ncr-1(nr2022); ncr-2: ncr-2(nr2023). * p<0.01. Error bar: sem; n>300. (TIF) [file pone.0033962.s003.tif]

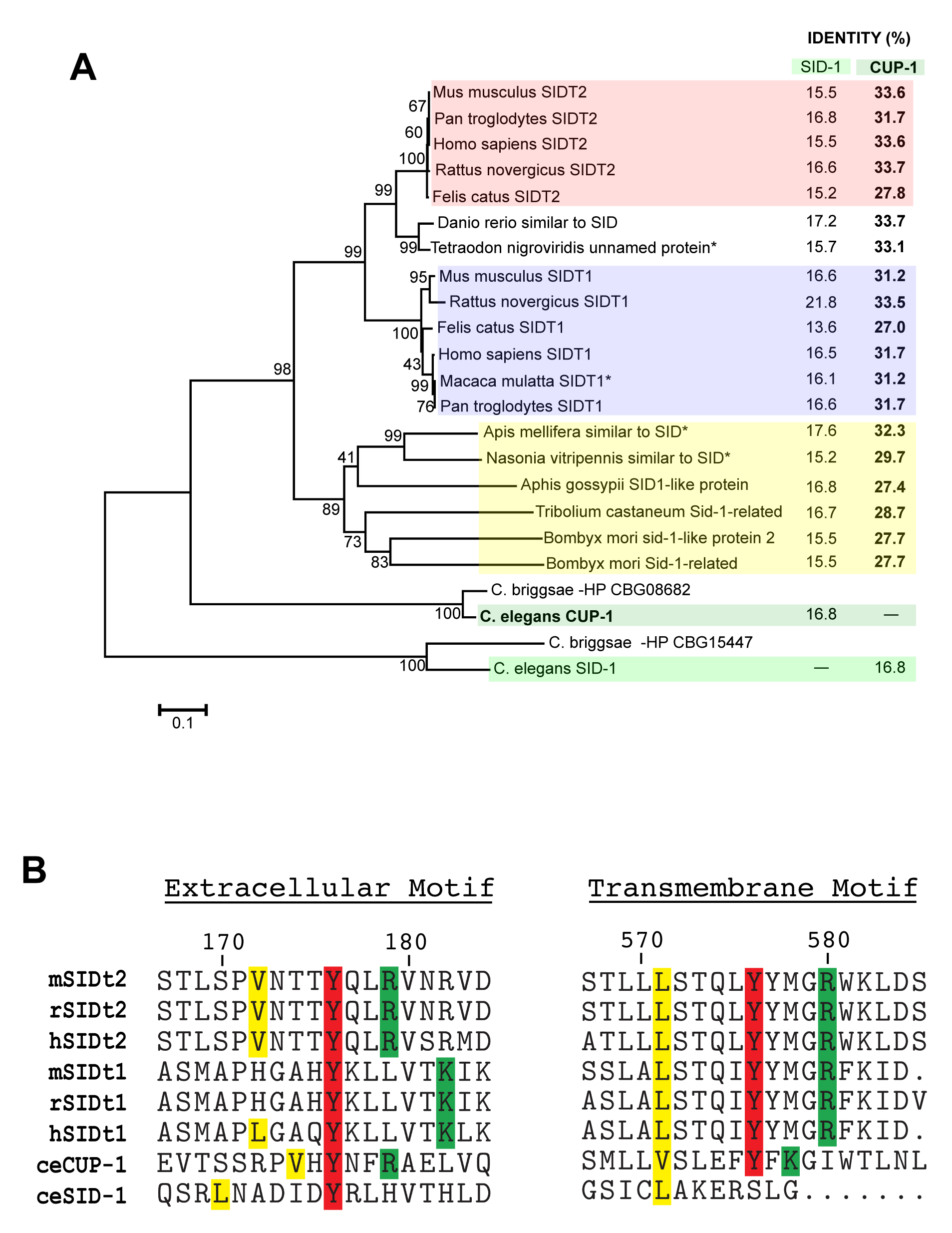

Supplement: Figure S4 — Phylogenetic analysis of CUP-1 homologue genes. Protein sequences of C. elegans CUP-1 homologues were aligned by ClustalW (MEGA5 [59]). (A) The multiple sequence alignment was then used to generate a phylogenetic tree by a maximum likelihood method using a WAG substitution model. Percentage of amino acid identity vs C. elegans SID-1 and CUP-1 are presented on the right. Numbers in branches indicate bootstrap values. (B) Alignment of the conserved extracellular and transmembrane CRAC motifs (L/V-X(1–5)-Y-X(1–5)-R/K) in CUP-1 homologue proteins. Numbers indicate amino acid position in CUP-1. m: Mus musculus; r: Rattus norvegicus; h: Homo sapiens; ce: Caenorhabditis elegans. (TIF) [file pone.0033962.s004.tif]
